# Analgesic Effect of SH003 and *Trichosanthes kirilowii* Maximowicz in Paclitaxel-Induced Neuropathic Pain in Mice

**DOI:** 10.3390/cimb44020050

**Published:** 2022-01-31

**Authors:** Ji Hwan Lee, Bonglee Kim, Seong-Gyu Ko, Woojin Kim

**Affiliations:** 1 Department of Physiology, College of Korean Medicine, Kyung Hee University, Seoul 02447, Korea; mibdna@khu.ac.kr; 2 Korean Medicine-Based Drug Repositioning Cancer Research Center, College of Korean Medicine, Kyung Hee University, Seoul 02447, Korea; bongleekim@khu.ac.kr (B.K.); epiko@khu.ac.kr (S.-G.K.)

**Keywords:** Allodynia, chemotherapy-induced neuropathic pain, Cucurbitacin D, Paclitaxel, *Trichosanthes* *kirilowii* Maximowicz

## Abstract

Pacliatxel is a taxol-based chemotherapeutic drug that is widely used to treat cancer. However, it can also induce peripheral neuropathy, which limits its use. Although several drugs are prescribed to attenuate neuropathies, no optimal treatment is available. Thus, in our study, we analyzed whether SH003 and its sub-components could alleviate paclitaxel-induced neuropathic pain. Multiple paclitaxel injections (cumulative dose 8 mg/kg, i.p.) induced cold and mechanical allodynia from day 10 to day 21 after the first injection in mice. Oral administration of SH003, an herbal mixture extract of *Astragalus membranaceus*, *Angelica gigas*, and *Trichosanthes* *kirilowii* Maximowicz (Tk), dose-dependently attenuated both allodynia. However, when administered separately only Tk decreased both allodynia. The effect of Tk was shown to be mediated by the spinal noradrenergic system as intrathecal pretreatment with α_1_- and α_2_-adrenergic-receptor antagonists (prazosin and idazoxan), but not 5-HT_1/2_, and 5-HT_3_-receptor antagonists (methysergide and MDL-72222) blocked the effect of Tk. The spinal noradrenaline levels were also upregulated. Among the phytochemicals of Tk, cucurbitacin D was shown to play a major role, as 0.025 mg/kg (i.p.) of cucurbitacin D alleviated allodynia similar to 500 mg/kg of SH003. These results suggest that Tk should be considered when treating paclitaxel-induced neuropathic pain.

## 1. Introduction

Paclitaxel, from the bark of *Taxus brevifolia*, is a widely used anti-cancer drug that has been used to treat breast, ovarian, and lung cancer [1,2,3]. Despite its well-known anti-cancer efficacy, the use of paclitaxel may be limited due to a severe dose-limiting factor, which is peripheral neuropathic pain [4]. It has been reported that within 24 h of its first administration, neuropathic pain occurs, characterized by cold and mechanical allodynia and numbness in the extremities [5,6]. This dose-limiting side effect may prevent the continuation of chemotherapy and also reduce the patient’s quality of life [7]. Furthermore, as cancers usually occur in aging patients [8], chemotherapy-induced pain management is important as untreated pain in the elderly is reported to be related with poor sleep, social isolation, functional deterioration and increased risk of falls [9,10]. Thus, a drug that can effectively alleviate chemotherapy-induced neuropathic pain (CIPN) is critically needed [11].

Anticonvulsants and antidepressants have been suggested as first-line treatments for neuropathic pain, such as gabapentin, serotonin- and noradrenaline- reuptake inhibitors (SNRI), respectively [12,13,14]. In particular, serotonin and noradrenaline are widely known for their involvement in pain [15]. Modulation of these neurotransmitters resulted in pain reduction in various clinical trials [15,16,17] and animal studies [18,19]. In CIPN as well, the role of serotonin and noradrenaline was shown to be critical as duloxetine, an SNRI, was recommended as the only agent for the treatment of CIPN by the recently released American Society of Clinical Organization guidelines [20]. Thus, in paclitaxel-induced neuropathic pain, modulating the serotonergic and noradrenergic systems could be an effective way to alleviate the severe cold and mechanical allodynia induced by paclitaxel injection. 

For many years, our group has been focusing on CIPN to understand its underlying mechanism of action and to find an effective drug [19,21,22,23,24]. In particular, we demonstrated that the analgesic effect of oxaliplatin-induced pain could be mediated by activation of the central descending pain-inhibitory system, such as the serotonergic and noradrenergic pathways [19,22,24]. In addition, we have also reported that serotonin and noradrenaline receptors present in the spinal cord play important roles in the treatment of pain [19,22,23,25]. Inhibition of the function of spinal receptors by intrathecal administration of their antagonists resulted in the failure of the analgesic effect induced by various treatments. We have demonstrated the analgesic effect of bee venom, morphine, and herbal medicines [22,26,27,28,29]. Among them, we focused on various herbal medicines, since herbal medicines are mostly known to be relatively safe and to induce only a low occurrence of side effects compared to other chemically modified drugs [30,31]. 

SH003 is a complex formula composed of an ethanol extract of three medicinal herbs, namely *Astragalus membranaceus*, *Angelica gigas*, and *Trichosanthes* *kirilowii* Maximowicz [32]. Previous studies have reported its anti-cancer effects on various types of cancer cells, such as breast, lung, and prostate cancer [33,34,35]. According to a recent study, the combination of SH003 and docetaxel, a chemotherapeutic agent, resulted in a synergetic anti-cancer effect [34]. Likewise, co-treatment with SH003 and paclitaxel caused apoptosis in paclitaxel-resistant breast-cancer cells [36], suggesting that co-treatment with SH003 and paclitaxel could result in synergism. Thus, if SH003 could alleviate the side effects caused by paclitaxel and increase the anti-cancer activity of paclitaxel, a combination of the two drugs will become one of the best options available. However, the analgesic mechanisms of SH003 on paclitaxel have not yet been assessed. Among the three components of SH003, *Astragalus membranaceus* has been reported to modulate serotonin and noradrenaline levels in the brain [37,38]. Therefore, we may assume that SH003 and its components could suppress paclitaxel-induced neuropathic pain via serotonin or noradrenaline modulation. 

## 2. Materials and Methods

### 2.1. Animals

Six-week-old C57BL/6 mice were obtained from Daehan Biolink (Chungbuk, Korea) and housed in a specific pathogen-free animal center. Mice were arbitrarily allocated to cages and were housed in a room with a temperature of 23 ± 2 ℃, humidity of 65 ± 5%, fixed 12 h light/dark cycle, and with food and water ad libitum. Before all experiments, mice were left untreated for one week to acclimate to the testing environments. All experimental protocols were approved by the Kyung Hee University Animal Care and Use Committee (KHUASP(SE)-20-448) on 15 November 2020. All experimental procedures were conducted in accordance with the guidelines of the International Association for the Study of Pain [39,40].

### 2.2. Administration of Paclitaxel

**Figure 1:**
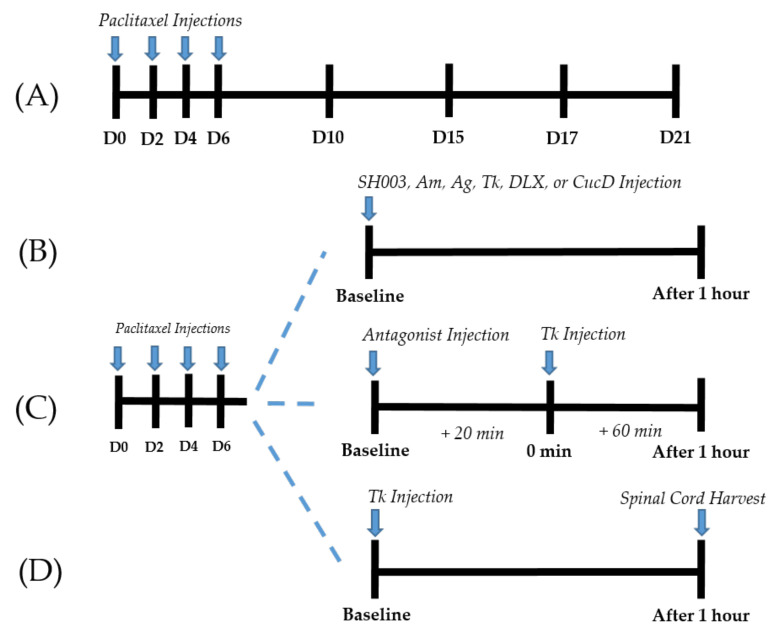
Experimental protocols (**A**–**D**). Timeline of behavioral tests conducted for paclitaxel-induced cold and mechanical allodynia assessments (**A**). Behavioral tests were conducted before (D0), 10 (D10), 15 (D15), 17 (D17), and 21 (D21) days following the first day of intraperitoneal administration of paclitaxel. Timeline of behavioral tests conducted to assess the effect of SH003, Am, Ag, Tk, DLX, and CucD (**B**). Behavioral tests were conducted before (baseline) and one hour after (After 1 h) the injection of SH003, Am, Ag, Tk, DLX, or CucD in paclitaxel-induced neuropathic pain mice. Timeline of behavioral tests conducted to clarify the role of spinal serotonergic and adrenergic receptors in Tk induced anti-allodynic effect (**C**). Antagonists were injected intrathecally 20 min before (baseline) the oral administration of Tk (0 min). Behavioral measurements were conducted twice; before the injection of antagonists (baseline) and one hour after Tk administration (After 1 h). Schedule for the ELISA assay (**D**). The L4/L5 segments of the spinal cord of mice were collected one hour after (After 1 h) the injection of Tk in paclitaxel-induced neuropathic pain mice.

Paclitaxel was prepared according to previous studies [23,41]. In brief, paclitaxel (Sigma Aldrich, St. Louis, MO, USA) was dissolved in 50% ethanol and 50% Cremophor EL solution to a concentration of 6 mg/mL. Prior to injection, paclitaxel was diluted in phosphate-buffered saline (PBS) to obtain a final concentration of 0.2 mg/mL. An equivalent concentration of solute was used as the vehicle. Paclitaxel (2 mg/kg) or vehicle was injected intraperitoneally (i.p.) every other day for a total of four injections (days 0, 2, 4, and 6). In total, 8 mg/kg paclitaxel were injected to induce cold and mechanical allodynia (Figure 1A).

### 2.3. Behavioral Tests

All behavioral tests were conducted as described in our previous study [21,22]. Cold and mechanical allodynia were measured and quantified using acetone-drop and von-Frey-filament tests, respectively. The caged animals were acclimatized in an inverted clear plastic cage (12 cm × 8 × 6 cm^3^) with a metal mesh floor for 30 min before all behavioral tests. 

For cold allodynia quantification, an acetone drop (10 μL) was applied to the mid-plantar hind paw of the mice. Behavioral responses (flicking and licking) against an acetone drop were observed and counted for 30 s. Thus, the term “# of Response” mentioned in the figures represents the average number of responses to an acetone drop of 10 μL, counted for 30 s.

To measure mechanical allodynia, a series of von Frey filaments (bending forces of 0.02, 0.04, 0.07, 0.16, 0.4, 0.6, 1, 1.4, and 2 g, Stoelting, Kiel, WI, USA) were applied to the mid-plantar hind paws. According to Dixon’s up-down method and Chaplan’s calculation method, a 50% threshold value was calculated [42,43]. The results are presented as the average of both hind paws.

### 2.4. Preparation and Treatment of SH003 and Its Components

SH003, *Astragalus* *membranaceus* (Am), *Angelica gigas* (Ag), and *Trichosanthes* *kirilowii* Maximowicz (Tk) were provided by HANPOONG (HANPOONG PHARM & FOODS Co., Jeonju, Korea) which followed good-manufacturing-practice (GMP) procedures. SH003 was prepared as described in a previous study [44]. Am, Ag, and Tk in a 1:1:1 ratio (*w*/*w*) were mixed and extracted with 30% ethanol up to approximately ten times the herb mixture at 100 °C for 3 h. The extract solution was concentrated by decompression at 60 °C [45]. Am and Ag were extracted with boiling water (105 °C) up to approximately ten times the herb mixture for 3 h. The extract solution was then concentrated by decompression at 60 °C Tk was extracted with 70% ethanol up to approximately ten times the herb mixture at 100 °C for 3 h. The extract solution was then concentrated by decompression at 60 °C. For oral administration, the extracts were diluted in PBS at a concentration of 50 mg/mL.

### 2.5. Administration of Serotonergic and Noradrenergic Antagonists

The involvement of spinal serotonergic receptors in the anti-allodynic efficacies of Tk against paclitaxel-induced neuropathic pain was assessed by intrathecal injection of serotonin-receptor antagonists. Injection of antagonists was conducted 20 min before the oral administration of Tk. The 5-HT-receptor antagonists used in the experiments were methysergide (mixed 5-HT_1/2_-receptor antagonists, 10 μg, concentration 2 μg/μL in PBS) and MDL-72222 (3-tropanyl-3,5-dichlorobenzoate, 5-HT_3_-receptor antagonist, 15 μg, concentration 3 μg/μL at 20% dimethyl sulfoxide). All antagonists used in this study were purchased from Tocris (Cookson, UK). For intrathecal injection, 5 μL of antagonist solution was injected at the lumbar 5–6 intervertebral level using a Hamilton syringe (Hamilton Company, Reno, NV, USA) under isoflurane anesthesia (2.5% in N_2_O/O_2_: 1:1 *v*/*v*) [19]. For the control, 5 μL of the solvent (PBS or 20% DMSO) was administered accordingly.

To examine whether adrenergic receptors at the spinal-cord level mediate the analgesic effects of Tk in paclitaxel-induced neuropathies, α_1_ and α_2_ antagonists were administered intrathecally 20 min prior to Tk oral treatment (Figure 1C). Prazosin (α_1_-adrenergic-receptor antagonist, 10 μg, 2 μg/μL in PBS) and idazoxan (α_2_-adrenergic-receptor antagonist, 10 μg, 2 μg/μL in PBS) were used in the experiments. Intrathecal injection of an adrenergic-receptor antagonist was performed using the same method as for serotonin-receptor-antagonist injection.

### 2.6. Enzyme-Linked Immunosorbent Assay (ELISA)

To determine whether Tk administration affects the levels of noradrenaline in the spinal cord, the levels of noradrenaline were measured by ELISA. After transcardial perfusion with 0.1 M PBS, the spinal cord from the animal was harvested via laminectomy one hour after Tk administration (Figure 1D). The L4/L5 segments of the spinal cord were identified by tracing the dorsal roots of the dorsal-root ganglia. Collected tissues were homogenized with 1 mL Pierce RIPA buffer (Thermo Scientific, Waltham, MA, USA) containing Halt protease and phosphatase inhibitor cocktail (Thermo Scientific, Waltham, MA, USA). Spinal cord samples were assayed using a commercial noradrenaline ELISA kit (Abnova, Neihu District, Taipei City, Taiwan) following the manufacturer’s protocol. Optical density (OD) was measured at 450 nm with a correction of 620 nm using a SOFT max PRO microplate reader (Molecular Devices, Sunnyvale, CA, USA). Total amounts of protein in the spinal cords were quantified using a Bio-Rad protein assay kit (Bio-Rad, Hercules, CA, USA). Samples and standards were run in duplicate. All recorded OD results were normalized to the total amount of protein in each sample.

### 2.7. Statistical Analysis

All data are presented as the mean ± standard error of the mean (SEM). Statistical analysis and graphic work were performed using Prism 7.0 (GraphPad software, La Jolla, CA, USA). Two-way analysis of variance (ANOVA) followed by Sidak’s post-test for multiple comparisons and one-way ANOVA followed by Tukey’s post-hoc test for multiple comparisons were used for statistical analyses. In all cases, *p* < 0.05 was considered to indicate significant differences.

## 3. Results

### 3.1. Paclitaxel-Induced Cold and Mechanical Allodynia in Mice

**Figure 2:**
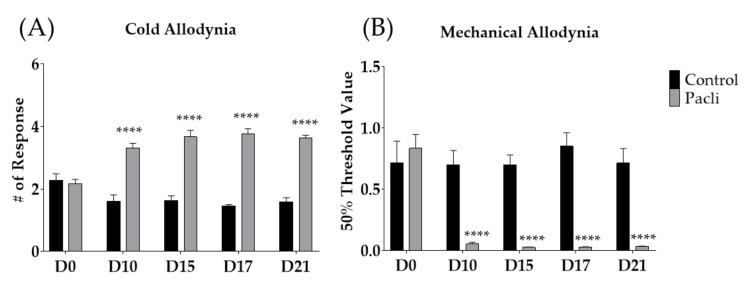
Effect of multiple paclitaxel (Pacli) injections on the response to cold and mechanical stimuli in mice (**A**,**B**). Paclitaxel was administered intraperitoneally on D0, D2, D4, and D6 (2 mg/kg each). Cold and mechanical allodynia were measured by using acetone-drop and von-Frey-filament tests, respectively. Behavioral tests were conducted before (D0) and 10, 15, 17, and 21 days following the initiation of paclitaxel treatment. Control-group mice were injected with a mixture of 50% ethanol and 50% Cremophor EL solution (1:1) as a control of paclitaxel (i.p.). Control: *n* = 7, Pacli: *n* = 8. **** *p* < 0.0001 vs. Control with two-way ANOVA followed by Sidak’s post-test for multiple comparisons.

Behavioral changes against cold and mechanical stimuli were recorded before (D0), 10 (D10), 15 (D15), 17 (D17), and 21 (D21) days following the first day of intraperitoneal administration of paclitaxel (Figure 2A,B). After multiple paclitaxel injections (D0, D2, D4, and D6), both cold and mechanical allodynia occurred significantly from D10 to D21 compared to the control group. These results are in accordance with those of our previous study [23]. Based on these results, all subsequent experiments were conducted between D10 and D21, when cold and mechanical allodynia were significantly induced in mice. 

### 3.2. Single Oral Administration of SH003 Alleviated Paclitaxel-Induced Cold and Mechanical Allodynia Dose-Dependently in Mice

**Figure 3:**
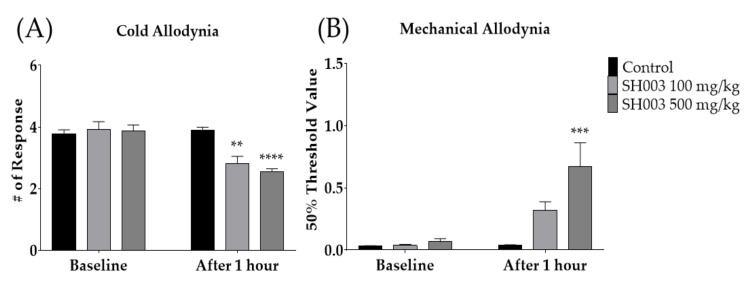
Effect of SH003 oral administration on cold and mechanical allodynia induced by multiple paclitaxel injection in mice (**A**,**B**). Behavioral measurements were conducted before (baseline) and 1 h after the treatment of SH003 or PBS. Two different doses of SH003 (100 and 500 mg/kg) and PBS were given orally. PBS was used as control to SH003. Control: *n* = 6, SH003 100 mg/kg: *n* = 6, SH003 500 mg/kg: *n* = 7. ** *p* < 0.01, *** *p* < 0.001, **** *p* < 0.0001 vs. Control with two-way ANOVA followed by Sidak’s post-test for multiple comparisons.

To elucidate whether SH003, a decoction of medicinal-herb formula, could alleviate paclitaxel-induced neuropathic pain, two different doses of SH003 (100 and 500 mg/kg) were orally administered to the mice. Behavioral changes were observed 1 h after SH003 administration. The results showed that paclitaxel-induced cold allodynia was significantly suppressed by both doses of SH003 (Figure 3A), whereas in mechanical allodynia, only 500 mg/kg of SH003 was effective (Figure 3B). These anti-allodynic effects of SH003 against paclitaxel-induced neuropathic pain were not shown 3 h after its treatment (data not shown).

### 3.3. Hydroalcoholic Extract of Trichosanthes kirilowii Maximowicz (Tk) Mediated the Anti-Allodynic Effect of SH003

**Figure 4:**
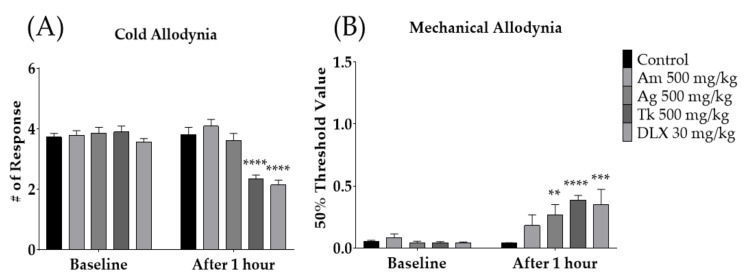
Effect of SH003 components on paclitaxel-induced cold and mechanical allodynia (**A**,**B**). Behavioral tests were conducted before and 1 h after the oral treatment of Am, As, Tk, DLX and PBS in mice. PBS was administered as a control to Am, Ag, Tk, and DLX. DLX was intraperitoneally injected as a positive control. Am: *Astragalus membranaceus*, Ag: *Angelica gigas*, Tk: *Trichosanthes kirilowii* Maximowicz, DLX: Duloxetine. Control: *n* = 13, Am 500 mg/kg: *n* = 6, Ag 500 mg/kg: *n* = 6, Tk 500 mg/kg: *n* = 6, DLX 30 mg/kg: *n* = 6. ** *p* < 0.01, *** *p* < 0.001, **** *p* < 0.0001 vs. Baseline with two-way ANOVA followed by Sidak’s post-test for multiple comparisons.

To evaluate which SH003 components mediate the analgesic effect of SH003, the anti-allodynic effect of *Astragalus membranaceus* (Am), *Angelica gigas* (Ag), and Tk were assessed accordingly. The degree of cold and mechanical allodynia was measured before and after the administration of 500 mg/kg of each medicinal herb, PBS, and duloxetine (DLX). PBS and DLX were used as the control and positive controls, respectively. The results showed that only Tk (500 mg/kg) and DLX, but not Am or Ag, significantly alleviated cold allodynia (Figure 4A). In mechanical allodynia, Ag, Tk, and DLX, but not Am, were effective (Figure 4B). As only Tk was effective against both cold and mechanical allodynia induced by paclitaxel, subsequent experiments were conducted using Tk. 

### 3.4. Intrathecal Administration of Serotonergic Receptors Antagonists Failed to Block the Analgesic Action of Tk in Mice

**Figure 5:**
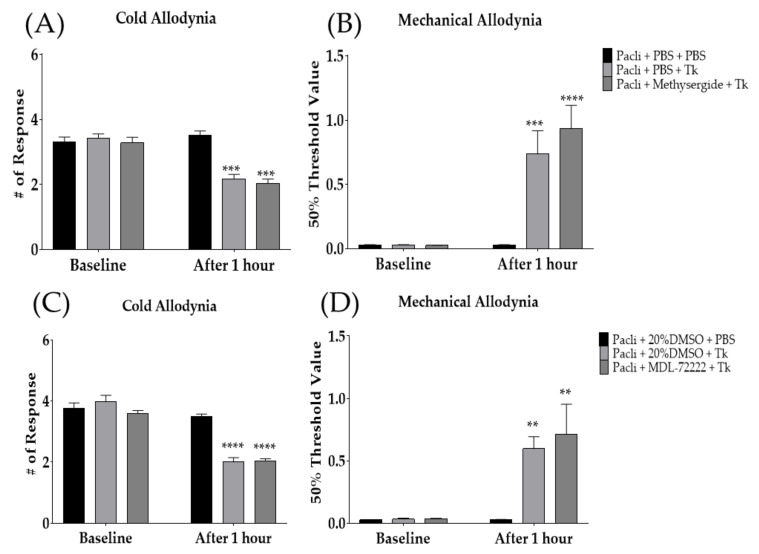
Analgesic effect of Tk following intrathecal injection of methysergide (**A**,**B**) or MDL-72222 (**C**,**D**) was assessed on cold (**A**,**C**) and mechanical (**B**,**D**) allodynia. Methysergide and MDL-72222 are 5-HT_1/2_ and 5-HT_3_-receptor antagonists, respectively. PBS and 20% DMSO were administered intrathecally as a control to methysergide and MDL-72222, respectively. Pacli: paclitaxel, Tk: *Trichosanthes kirilowii* Maximowicz. Pacli + PBS + PBS: *n* = 6, Pacli + PBS + Tk: *n* = 6, Pacli + methysergide + Tk: *n* = 6, Pacli + 20%DMSO + PBS: *n* = 6, Pacli + 20%DMSO + Tk: *n* = 6, Pacli + MDL-72222 + Tk: *n* = 6. ** *p* < 0.01, *** *p* < 0.001, **** *p* < 0.0001 vs. baseline with two-way ANOVA followed by Sidak’s post-test for multiple comparisons.

To investigate whether spinal serotonergic receptors are involved in the anti-allodynic effect of Tk against paclitaxel-induced allodynia, serotonergic-receptor antagonists, methysergide and MDL-72222 were injected intrathecally 20 min prior to oral administration of Tk. Methysergide and MDL-72222 are 5-HT_1/2_- and 5-HT_3_-receptor antagonists, respectively. In our previous studies, both methysergide and MDL-72222 did not affect the degree of cold and mechanical allodynia in mice [22,24]. The results demonstrated that both 5-HT_1/2_- and 5-HT_3_-receptor antagonists failed to block the analgesic effect of Tk, as Tk significantly attenuated both cold (Figure 5A,C) and mechanical (Figure 5B,D) allodynia at 1 h after its treatment. These results suggest that spinal serotonergic receptors may not be involved in the anti-allodynic effect of Tk. 

### 3.5. Anti-Allodynic Effect of Tk Was Mediated by Spinal Noradrenergic System

**Figure 6:**
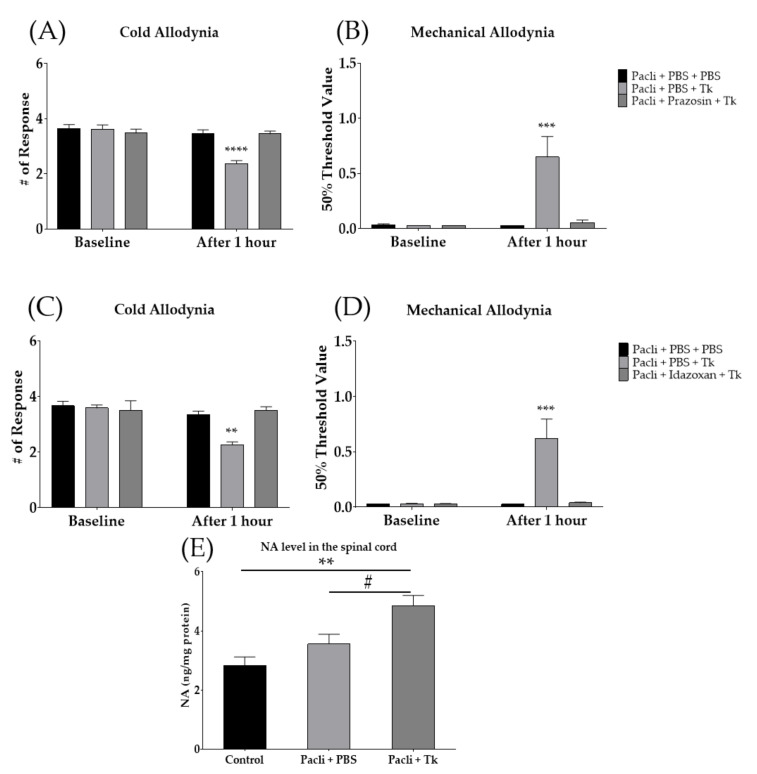
Involvement of spinal noradrenergic system in the anti-allodynic effect of Tk. The effect of intrathecally injected prazosin (α_1_-adrenergic-receptor antagonist) (**A**,**B**) and idazoxan (α_2_-adrenergic-receptor antagonist) (**C**,**D**) on the effect of Tk. Changes in noradrenaline (NA) contents in the spinal cord following 1 h of Tk (500 mg/kg) oral administration (**E**). PBS was used as a control to prazosin, idazoxan and Tk (**A**–**E**). Control is mixture of 50% EtOH and 50% Cremophor EL solution (1:1) treated group as a control of Pacli + PBS (**E**). Pacli: paclitaxel, Tk: *Trichosanthes kirilowii* Maximowicz. Pacli + PBS + PBS: *n* = 6, Pacli + PBS + Tk: *n* = 6, Pacli + prazosin + Tk: *n* = 6, Pacli + PBS + PBS: *n* = 6, Pacli + PBS + Tk: *n* = 6, Pacli + Idazoxan + Tk: *n* = 6 (**A**–**D**). ** *p* < 0.01, *** *p* < 0.001, **** *p* < 0.0001 vs. baseline with two-way ANOVA followed by Sidak’s post-test for multiple comparisons. Control: *n* = 6, Pacli + PBS: *n* = 6, Pacli + Tk: *n* = 6 (**E**). ** *p* < 0.01 vs. Control and # *p* < 0.05 vs. Pacli + PBS with one-way ANOVA followed by Tukey’s post-test for multiple comparisons.

Since it was found that the spinal serotonergic system did not take part in the analgesic effect of Tk, we subsequently focused on the spinal noradrenergic system. Noradrenergic-receptor antagonists were injected intrathecally before the Tk treatment. Prazosin and idazoxan are α_1_- and α_2_-adrenergic-receptor antagonists, respectively. The results showed that, in contrast to the serotonergic-receptor antagonist, both spinal adrenergic-receptor antagonists blocked the effect of Tk on paclitaxel-induced cold (Figure 6A,C) and mechanical (Figure 6B,D) allodynia. Furthermore, to assess whether the spinal noradrenaline level is affected by oral administration of Tk, ELISA was conducted accordingly. One hour after oral treatment with 500 mg/kg of Tk, noradrenaline (NA) content in the spinal cord was significantly increased compared to the control group and Pacli + PBS group. NA levels were not altered after paclitaxel injection (Figure 6E). 

### 3.6. Cucurbitacin D Mimicked the Anti-Allodynic Effect of Tk in Allodynia Induced Mice 

**Figure 7:**
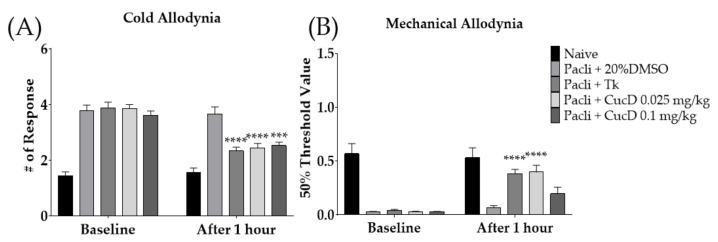
Efficacy of cucurbitacin D (CucD), a component of Tk, on paclitaxel-induced neuropathic pain (**A**,**B**). Efficacy of Tk (500 mg/kg) and CucD (0.025 and 0.1 mg/kg) were investigated 1 h after its treatment. 20% DMSO was used as a control of CucD (i.p.). Tk was orally administered as a positive control. CucD: Cucurbitacin D, Tk: *Trichosanthes kirilowii* Maximowicz. Naive: *n* = 6, Pacli + 20%DMSO: *n* = 6, Pacli + Tk: *n* = 6, Pacli + CucD 0.025 mg/kg: *n* = 6, Pacli + CucD 0.1 mg/kg: *n* = 6. *** *p* < 0.001, **** *p* < 0.0001 vs. Baseline with two-way ANOVA followed by Sidak’s post-test for multiple comparisons.

Cucurbitacin D (CucD) is one of the key phytochemicals present in Tk [46,47]. Thus, we conducted behavioral experiments using CucD. Two different doses of CucD (0.025 and 0.1 mg/kg) were intraperitoneally injected into allodynia-induced mice. The dose of CucD was determined based on a literature search; approximately 50 μg/g of CucD was included in the root of Tk [46]. Accordingly, 0.025 mg/kg of CucD was present in 500 mg/kg of Tk. To validate the anti-allodynic effects of CucD, behavioral changes were observed 1 h after the intraperitoneal injection of CucD. The results showed that Tk (500 mg/kg) and both doses of CucD (0.025 and 0.1 mg/kg) significantly lowered the number of responses to cold stimuli (Figure 7A). However, on mechanical allodynia, Tk and 0.025 mg/kg, but not 0.1 mg/kg of CucD showed an anti-allodynic effect (Figure 7B).

## 4. Discussion

In our study, we demonstrated that the oral administration of SH003 could significantly suppress paclitaxel-induced cold and mechanical allodynia in mice. Among the three components of SH003, Tk was shown to be the only component that could attenuate both the cold and mechanical allodynia induced by paclitaxel. Furthermore, the analgesic effect of Tk was shown to be mediated by spinal α_1_- and α_2_-adrenergic receptors, but not by serotonergic receptors, as intrathecal injection of adrenergic antagonists completely blocked the effect of Tk. The spinal noradrenalin level was also upregulated after oral treatment with Tk. Finally, we demonstrated that CucD may be the key molecule of Tk, since an intraperitoneal injection of 0.025 mg/kg of CucD significantly alleviated the pain in mice. To the best of our knowledge, this is the first study to demonstrate the anti-allodynic effects of SH003, Tk, and CucD in paclitaxel-induced neuropathic pain. 

Paclitaxel, along with its efficient anti-tumor effect, can cause severe pain, as reported in studies from other labs [4,5,6] and in our study (Figure 2A,B). Paclitaxel-induced neuropathic pain manifests as cold and mechanical allodynia in animal models [23,41]. As part of its pathological mechanism, it is known that accumulated paclitaxel in dorsal-root ganglia causes axon degeneration and demyelination of large- and small-fiber sensory neurons [48,49]. As a result, sensory input from damaged peripheral neurons can induce hyperactivation of spinal neurons, which finally develops into central sensitization [11,23]. Duloxetine, a serotonin–noradrenalin-reuptake inhibitor (SNRI), is known to be effective [12,13,14]; however, the use of these drugs is limited because of dose-limiting effects, such as sedation and reduced locomotive activity [50,51,52].

SH003 is a herbal formula comprising three medicinal herbs, *Astragalus membranaceus* (Am), *Angelica gigas* (Ag), and Tk. Accumulating evidence has demonstrated that SH003 has anti-cancer efficacy against various types of cancer [35,36,45], and clinical trials have been conducted to analyze the efficacy and safety of SH003 in patients with solid cancers [32,53]. The effect of SH003 in chemotherapy-induced neuropathic pain has been demonstrated in docetaxel-induced mechanical allodynia [54]; however, its effect on paclitaxel has never been investigated. Although both docetaxel and paclitaxel are taxanes, paclitaxel has been reported to be more neurotoxic than docetaxel [48]. Furthermore, in a previous study [54], the effect of SH003 components was not investigated, whereas in this study, the effect of each component was assessed accordingly. Our results showed that only Tk could significantly attenuate both cold and mechanical allodynia induced by paclitaxel injection. The effect of Tk was similar to 30 mg/kg of duloxetine (Figure 4A,B). 

Tk is a medicinal herb that has been used in China, Japan, and Korea. Tk is categorized in the genus *Trichosanthes*, family *Cucurbitaceae* [47]. The genus *Trichosanthes* is mainly distributed in eastern and southern Asia in warm and moist habitats [47,55]. As a medicinal use, it has been known that different parts of the plant, such as the fruit, root, and seed, have different efficacies [31]. In this study, the root tuber of Tk was used, which has been reported to have a treatment effect against diabetes [56]. However, to our knowledge, this is the first study to assess the effect of Tk on chemotherapy-induced neuropathic pain. Tk was shown to inhibit pain transmission in the spinal cord through spinal α_1_- and α_2_-adrenergic receptors, as selective antagonist pretreatment completely blocked the analgesic effect of Tk. However, serotonergic-receptor antagonists failed to block the effect of Tk. Furthermore, Tk significantly increased noradrenaline levels in the spinal cord, demonstrating that Tk attenuates pain by modulating the noradrenaline system in the central nervous system.

Noradrenaline and its receptors are part of the descending pain-inhibitory system, which starts from the brainstem and is distributed through the spinal cord [57]; under pain conditions, the activated descending noradrenergic system blocks pain transmission [58]. In accordance with the results obtained from this study, we previously reported the involvement of both α_1_- and α_2_-adrenergic receptors in the mechanisms of pain suppression [23,29,59]. The spinal α_1_-adrenergic receptor is known to be present in presynaptic terminals and the soma or dendrites of inhibitory interneurons in the substantia gelatinosa [60,61]. Noradrenaline stimulates α_1_-adrenergic receptors located in presynaptic terminals and γ-aminobutyric acid-ergic neurons, and suppresses the activity of wide-dynamic-range neurons, thus decreasing pain [62]. In addition, spinal pre- and postsynaptic α_2_-adrenergic receptors are coupled to the inhibitory G protein (Gi/o) [63]. Presynaptic α_2_-adrenergic receptors, located in primary nociceptive neurons, bind with noradrenaline, thus decreasing the secretion of excitatory neurotransmitters such as glutamate and substance P [63]. In secondary sensory neurons, activation of postsynaptic α_2_-adrenergic receptors results in the reduction in neuronal excitability via the opening of inwardly rectifying K+ channels [64]. Thus, Tk may increase the secretion of noradrenaline into the spinal cord, and noradrenaline may decrease the pain transmission through activation of its receptors present on spinal neurons.

A large portion of the root tuber of Tk is composed of various types of cucurbitacins, which are tetracyclic triterpenoids [46,47,58]. Among them, cucurbitacin B (approximately 250 μg/g) and CucD (approximately 50 μg/g) were present in the largest quantity [46]. In our study, we focused on CucD because of the cytotoxicity of cucurbitacin B and CucD, and anti-cancer effects in various cancer models have been reported in literature [65,66,67,68]. Despite the observed toxicity of both cucurbitacin B and CucD, the cell viability of endothelial cells of the pulmonary artery (CPAE) was measured after Tk extract inoculation to evaluate the safety of Tk, and Tk did not show any significant toxicity to CPAE (Supplementary Figure S1). 

The limitation of this study is that relatively young mice (6 weeks old) were used in order to mimic the allodynia present in paclitaxel-treated patients. Cancers usually develop in the elderly [8] and it is known that the biomarkers of aging are not detected until 15 months of age in mice [69]. Thus, it should be important in the next study to show the analgesic effect of SH003, Tk and CucD in 15-month-old paclitaxel-injected mice.

In conclusion, our results demonstrated that SH003 suppresses paclitaxel-induced cold and mechanical allodynia. Tk is an herbal component responsible for the anti-allodynic efficacy of SH003 via the spinal noradrenergic pathway. Among the components of SH003, Tk was the most effective. Thus, SH003 and Tk should both be considered as therapeutic agents for paclitaxel-induced neuropathy.

## 5. Patents

The content of this article is related to a patent application in Korea (10-2022-0004880).

## Data Availability

Not applicable.

## Supplementary Materials

The following supporting information can be downloaded at: https://www.mdpi.com/article/10.3390/cimb44020050/s1. Figure S1: Toxicity test of Tk on CPAE cell line.
