# Supplementary material for: Analgesic Effect of SH003 and Trichosanthes kirilowii Maximowicz in Paclitaxel-Induced Neuropathic Pain in Mice
Source: Curr Issues Mol Biol. 2022 Jan 31;44(2):718–30. doi: 10.3390/cimb44020050 (PMC8929024; doi:10.3390/cimb44020050)
Supplement: Supplementary file 1 [file cimb-44-00050-s001.zip › cimb-1510501-supplementary.pdf]

current issues in
molecular biology

<Supplementary Data>

Toxicity Test

Endothelial cell lines derived from the pulmonary artery (CPAE) were used to evaluate the toxicity of *Trhichosanthes kirilowii* Maximowicz (Tk). Toxicity assay was performed using the EZ-Cytox cell viability assay kit (Daeil Lab Service, Seoul, Korea) according to the manufacturer's instructions. Cells were seed and exposed to various concentrations of DD (6.25, 12.5, 25, 50, 100, 200, and 400 μg/mL) for 24 h onto 96 well plate. The cells were incubated with EZ-Cytox solution until formazan was formed for 1 hour. The absorbance values were measured at 450 nm using a microplate reader (Bio-Rad, Hercules, CA, USA). The CPAE cell line was cultured in RPMI 1640 medium (WELGENE, Kyungsan-si, Kyungbuk, Korea) and 10% fetal bovine serum (WELGENE, Kyungsan-si, Kyungbuk, Korea) supplemented with 1% penicillin (WELGENE, Kyungsan-si, Kyungbuk, Korea) and streptomycin (WELGENE, Kyungsan-si, Kyungbuk, Korea).

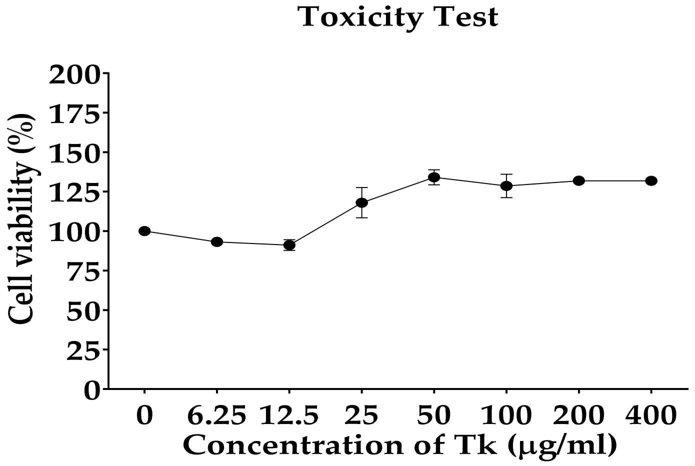

Supplementary Figure S1. Tk inoculation did not affect the cell viability of CPAE cell line.
